# A Conceptual Translational Bench-to-Bedside Cardiac Safety Framework for Predicting Drug-Induced QT Interval Prolongation from Molecular Structure to Clinical Data

**DOI:** 10.3390/ijms27209312

**Published:** 2026-10-20

**Authors:** Fianne Sips, Marc-Antonio Bisotti, Mario Torchia, Vincenzo Carbone, Nicola Gambacorta, Valentina Belgiovine, Fulvio Ciriaco, Orazio Nicolotti, Beatriz Trenor, Javier Saiz, Jari Hyttinen, Stefano Severi, Urban Fagerholm, Brian E. Carlson, Candice N. Baker, J Matthew Mahoney, Daniel Röshammar

**Affiliations:** 1 InSilicoTrials Technologies B.V., 1017 BR Amsterdam, The Netherlands; fianne.sips@insilicotrials.com; 2 InSilicoTrials Technologies S.p.A., 34123 Trieste, Italy; marc-antonio.bisotti@insilicotrials.com (M.-A.B.); mario.torchia@insilicotrials.com (M.T.); vincenzo.carbone@insilicotrials.com (V.C.); 3 Dipartimento di Farmacia—Scienze del Farmaco, Università degli Studi di Bari “Aldo Moro”, 70125 Bari, Italy; nicola.gambacorta1@uniba.it (N.G.); valentina.belgiovine@uniba.it (V.B.); orazio.nicolotti@uniba.it (O.N.); 4 Dipartimento di Chimica, Università degli Studi di Bari “Aldo Moro”, 70125 Bari, Italy; fulvio.ciriaco@uniba.it; 5 Centro de Investigación e Innovación en Bioingeniería (Ci2B), Universitat Politècnica de València, 46022 Valencia, Spain; btrenor@eln.upv.es (B.T.); jsaiz@ci2b.upv.es (J.S.); 6 Faculty of Biomedical Sciences and Engineering, Tampere University of Technology, FI-33720 Tampere, Finland; jari.hyttinen@tuni.fi; 7 Department of Electrical, Electronic and Information Engineering, University of Bologna, 47522 Cesena, Italy; stefano.severi@unibo.it; 8 Prosilico AB, SE-141 45 Huddinge, Sweden; urban.fagerholm@prosilico.com; 9 Department of Molecular & Integrative Physiology, University of Michigan, Ann Arbor, MI 48109, USA; bcarl@med.umich.edu; 10 The Jackson Laboratory, Bar Harbor, ME 04609, USA; candice.baker@jax.org (C.N.B.); matt.mahoney@jax.org (J.M.M.)

**Keywords:** cardiovascular safety, drug discovery and development, QT, molecular structure, in silico, in vitro, in vivo, clinical

## Abstract

QT interval prolongation is associated with an increased risk of torsades de pointes (TdP), a potentially life-threatening ventricular arrhythmia, making assessment of cardiac repolarization a critical component of drug development. This work demonstrates how integrated in silico methodologies can be used to predict and quantitatively characterize QT liability across the drug discovery and development continuum. A proof-of concept translational workflow combining molecular structure analysis, in vitro pharmacology, animal data, and clinical exposure-response modeling was evaluated using the retrospective case examples verapamil and dofetilide. Molecular structure- and pharmacology-based assessments predicted hERG channel interaction and potential QT risk for both compounds. While initial in silico analyses suggested a high QT liability for verapamil and dofetilide, mechanistic electrophysiological modeling differentiated their effects. For verapamil, concurrent calcium channel inhibition offset hERG-mediated effects, resulting in predicted reductions in action potential duration and no clinically relevant QT prolongation. In contrast, dofetilide produced concentration-dependent increases in action potential duration, consistent with its known proarrhythmic potential. Translation of nonclinical findings to humans accurately predicted the absence of clinically meaningful QT effects for verapamil and identified QT prolongation exceeding the regulatory 10 ms threshold for dofetilide at therapeutic concentrations. Drug exposure and pharmacokinetic properties could be anticipated based solely on molecular structure. This conceptual workflow demonstrates how integrated quantitative in silico approaches can support earlier identification, characterization, and communication of QT risk, enabling informed decision-making and improved development efficiency from bench to bedside.

## 1. Introduction

### 1.1. QT Assessment in Drug Development

QT interval prolongation is associated with an increased risk of torsades de pointes (TdP), a potentially life-threatening ventricular arrhythmia. Because of this risk, regulatory agencies require that virtually all new drug candidates, regardless of therapeutic area, undergo assessment of their potential effects on QT prolongation during clinical development. According to the International Council for Harmonisation (ICH) E14 guidelines, a drug is generally considered to raise regulatory concern if it causes a QT interval prolongation exceeding 10 ms (upper bound of the 90% confidence interval [CI]) at clinically relevant therapeutic drug exposure [1,2]. Compounds that exceed this threshold may require a comprehensive benefit–risk assessment, additional electrocardiogram (ECG) monitoring, or discontinuation of development.

In this context, early decision making is critical [3]. Drug candidates with a high proarrhythmic risk and an unfavorable benefit-risk profile should ideally be terminated before substantial investments are made in late-stage clinical programs. For candidates that continue in development, a thorough characterization of QT liability is essential, including quantitative exposure-response analyses and scientifically justified dose selection.

Following the publication of the white paper by Garnett et al., which introduced a standardized concentration–QT modeling framework, the clinical assessment of QT prolongation has substantially evolved. The traditional requirement for a dedicated thorough QT (TQT) study has largely been replaced by model-based analyses using ECG and drug concentration data collected in early-phase clinical studies, including single ascending dose (SAD) and multiple ascending dose (MAD) trials. This approach enables efficient characterization of QT effects while reducing the operational development burden [4].

In preclinical development, the potential for QT interval prolongation is commonly assessed using animal models, particularly rats and dogs. Translation from relevant non-rodent species, especially telemetered dogs, has been shown to predict QT findings in humans with 90% accuracy [5].

The Comprehensive In Vitro Proarrhythmia Assay (CiPA) was established to develop a new paradigm for assessing proarrhythmic risk. Within this framework, in vitro measurements of drug effects on cardiac ion channels are translated to clinical risk predictions using in silico methods. This is not limited to drug interaction with the potassium channel encoded by the ether-à-go-go related gene (hERG) but includes other ion channels such as calcium (Ca_v_1.2) and sodium (Na_v_1.5) [6,7].

Advances in computational chemistry and machine learning have also enabled the early prediction of QT liability based on molecular structure. Quantitative structure–activity relationship (QSAR) models have identified that amines, ethers, and aromatic moieties are commonly observed for drugs associated with QT prolongation but rarely in drugs with no apparent cardiac safety concern [8].

Overall, QT assessment during drug discovery and development encompasses two key objectives. First, it should provide evidence to support the investigation of new drugs tested in humans and to inform labeling decisions. Second, it facilitates the early-stage screening, prioritization, and optimization of new drug candidates, thereby helping to minimize late-stage attrition and enabling more efficient resource allocation.

Although a wide range of experimental and analytical methods are available to assess QT risk, these approaches are often applied in isolation and may require specialized expertise. The objective of this work is to conceptually demonstrate how integrated in silico methodologies and model-based approaches can be leveraged to predict QT liability and quantitatively characterize drug effects on cardiac repolarization throughout the drug development process. Specifically, we illustrate how proarrhythmic risk can be anticipated using molecular structure and in vitro pharmacology data as well as how QT effects observed in animal and clinical studies can be quantitatively estimated using unified model-based approaches. By establishing an accessible and scalable quantitative workflow, this proof-of-concept framework aims to support both technical specialists and multidisciplinary project teams, ultimately facilitating informed decision-making throughout the drug development process.

### 1.2. Cardioverse Hub

**Figure 1:**
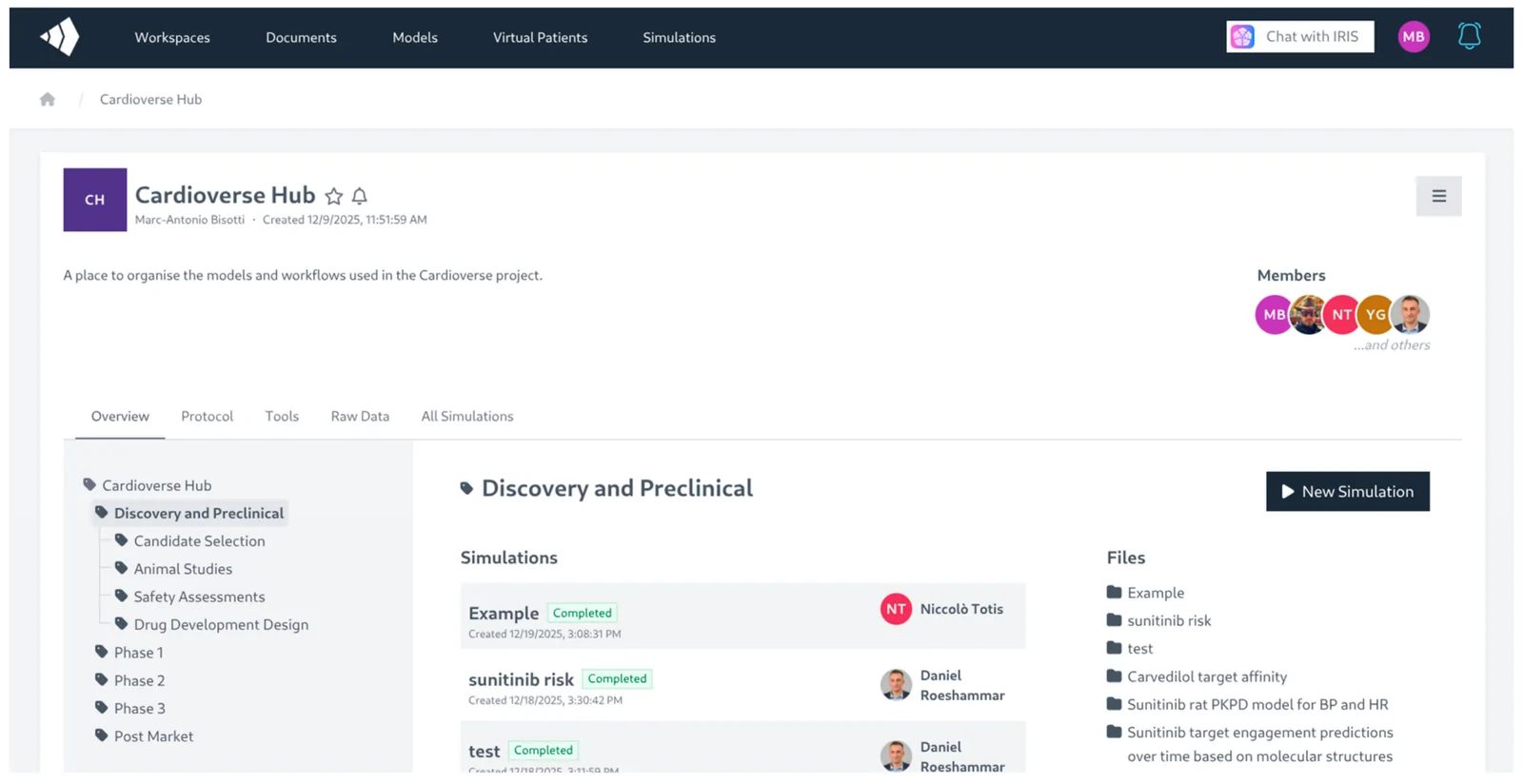
Overview of the Cardioverse hub. Various in silico solutions can be linked to inform different stages of drug development. The solutions can readily be reused across drug projects to harmonize objective and transparent decision-making.

The Cardioverse hub is a computational working environment designed to integrate diverse data sources, tools, and modeling approaches within a unified workflow for cardiovascular safety assessment. The Cardioverse hub is implemented on the InSilicoTrials platform, which hosts some of the applications described in this workflow. By bringing together molecular structure, in vitro, preclinical, and clinical data, the platform supports decision-making across all stages of drug development. It thus enables informed go/no-go decisions and optimized dose selection based on the totality of evidence (Figure 1).

**Figure 2:**
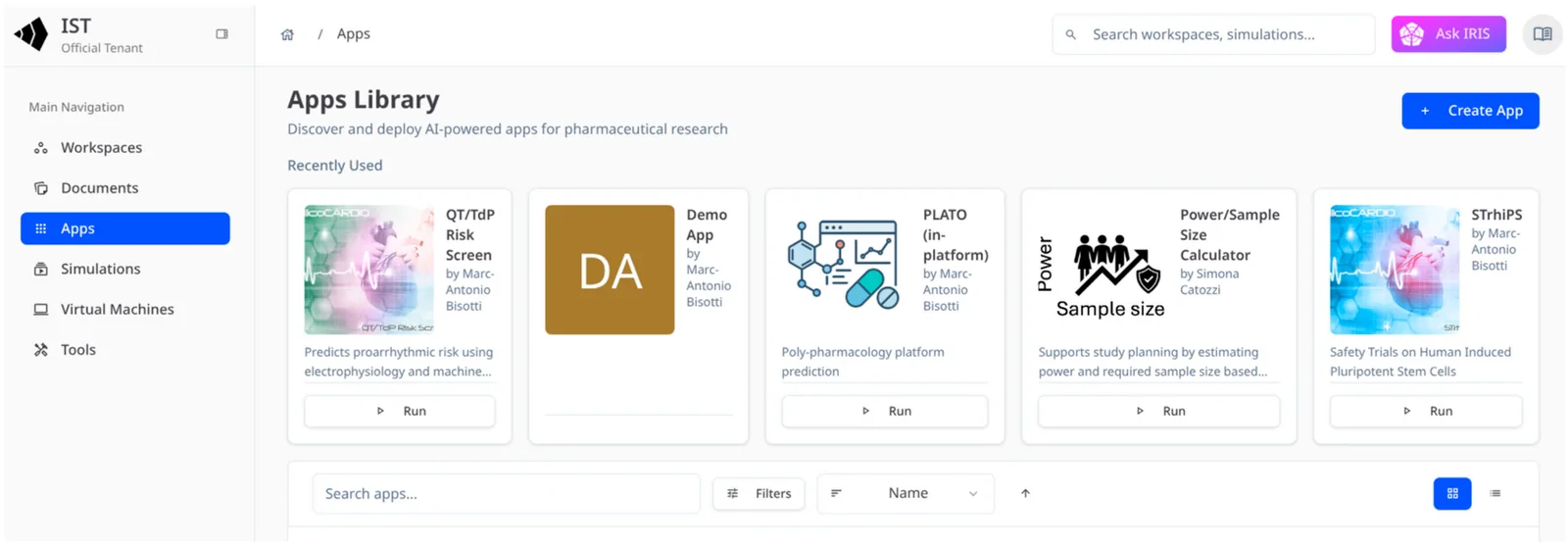
Applications library. Various internally and externally developed software, tools, and methods can be stored on the platform and integrated in various workflows. For each application, both the simulation input wizard and the results dashboard are fully customizable.

The Cardioverse hub incorporates a library of multiple applications designed to address a broad range of drug safety assessment questions commonly encountered throughout the drug development process. These applications leverage diverse data inputs, including molecular structure, in vitro pharmacology, preclinical, and clinical studies, to support integrated analyses within a unified computational framework (Figure 2).

## 2. Results

### 2.1. CUPID

**Figure 3:**
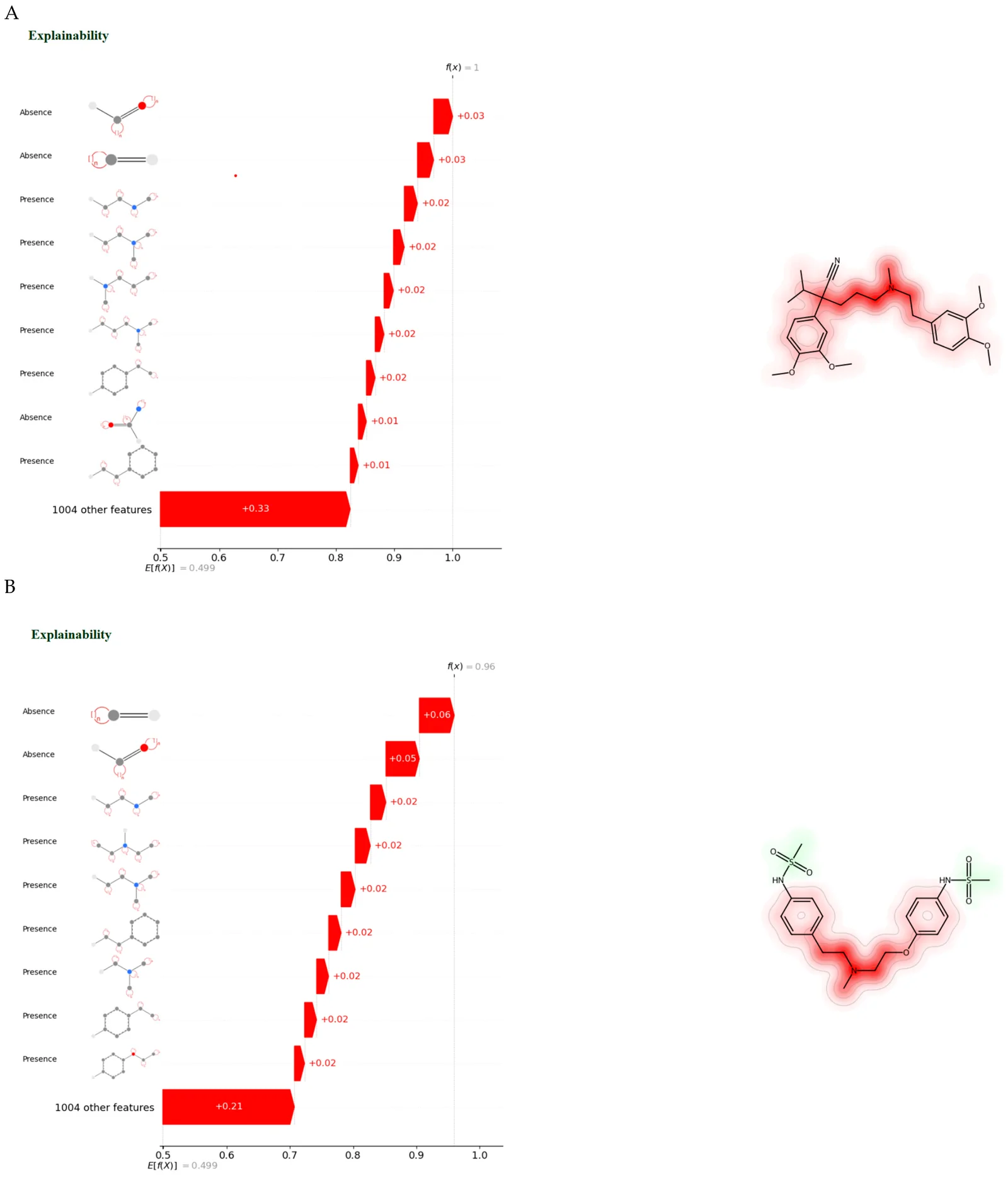
Results dashboard from the CUPID analysis of verapamil (**A**) and dofetilide (**B**) showing the structural parts of the molecule that are predicted to contribute to the cardiotoxicity risk on hERG interaction.

To obtain an early assessment of potential interactions with different ion channels, molecular structures can be screened using computational tools such as CUPID (Cardiotox Understanding Platform for Intelligent Drug discovery) [9]. For verapamil, CUPID predicted a high risk of interaction with hERG (prediction reliability of 94.4%) and Cav 1.2 (prediction reliability of 95.8%) but not Nav 1.5 with a prediction reliability of 84.1% based on structural chemical components of the molecule (Figure 3A). For dofetilide, CUPID predicted a high risk of interaction with hERG (prediction reliability of 94.4%) but not Cav 1.2 or Nav 1.5 with a prediction reliability of 73.7% and 84.1%, respectively (Figure 3B).

### 2.2. PLATO

**Figure 4:**
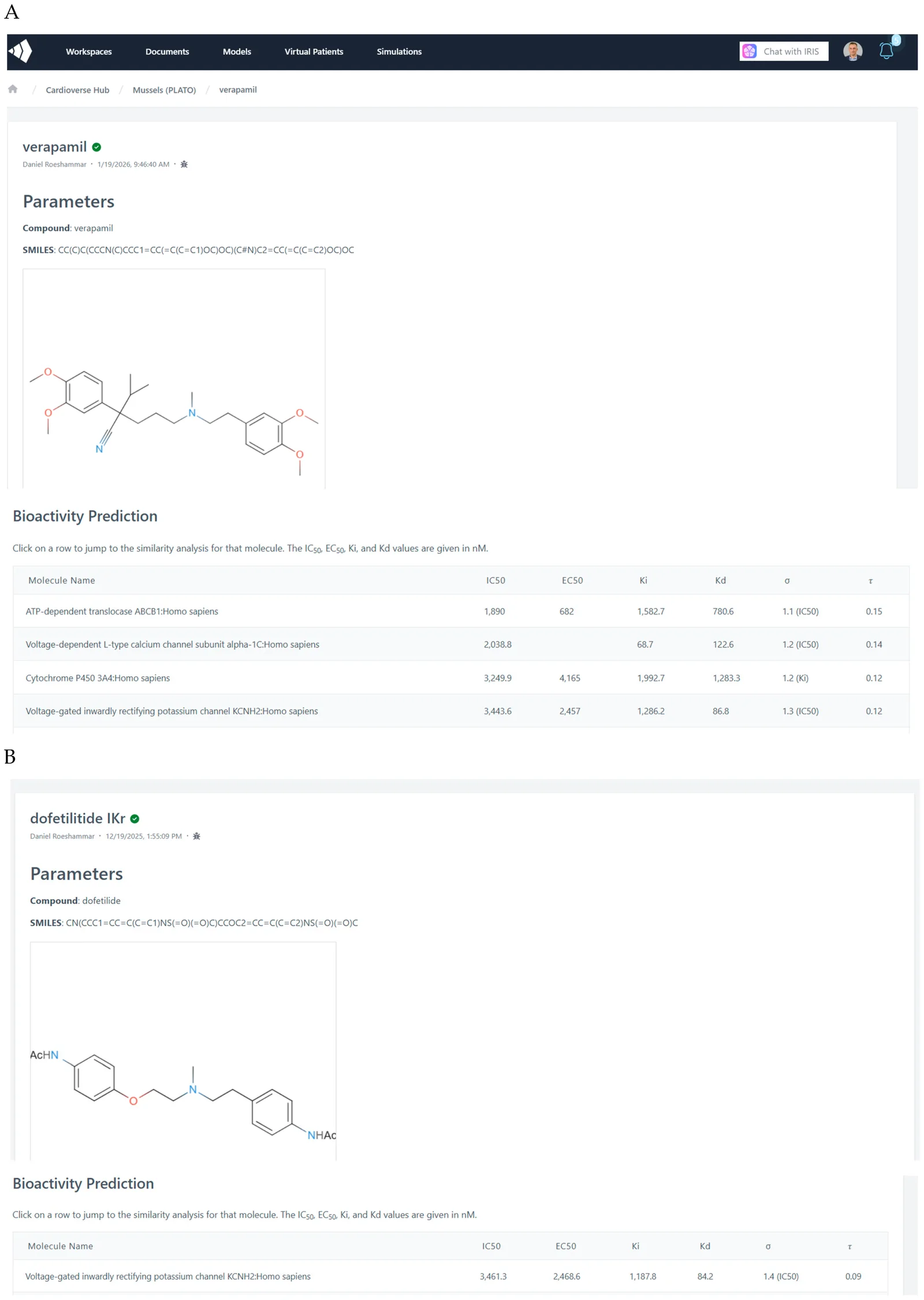
Results dashboard from PLATO bioactivity predictions for verapamil (**A**) and dofetilide (**B**) based on molecular structure.

Based on molecular structure, tools such as PLATO (Polypharmacology pLATform prediction, release 36, based on ChEMBL database version 36) can be used to screen for potential bioactivity against a broad range of biological targets, including ion channels, receptors, enzymes, and transporters [10,11,12,13]. For identified targets, drug affinity prediction is subsequently estimated. For verapamil, PLATO predicted bioactivity and estimated binding affinities for the expected Cav 1.2 and hERG channel interactions, which can be used as input for further assessment of QT risk (Figure 4A). For dofetilide, PLATO predicted bioactivity and provided binding affinities for the hERG channel interaction (Figure 4B).

### 2.3. ANDROMEDA

**Figure 5:**
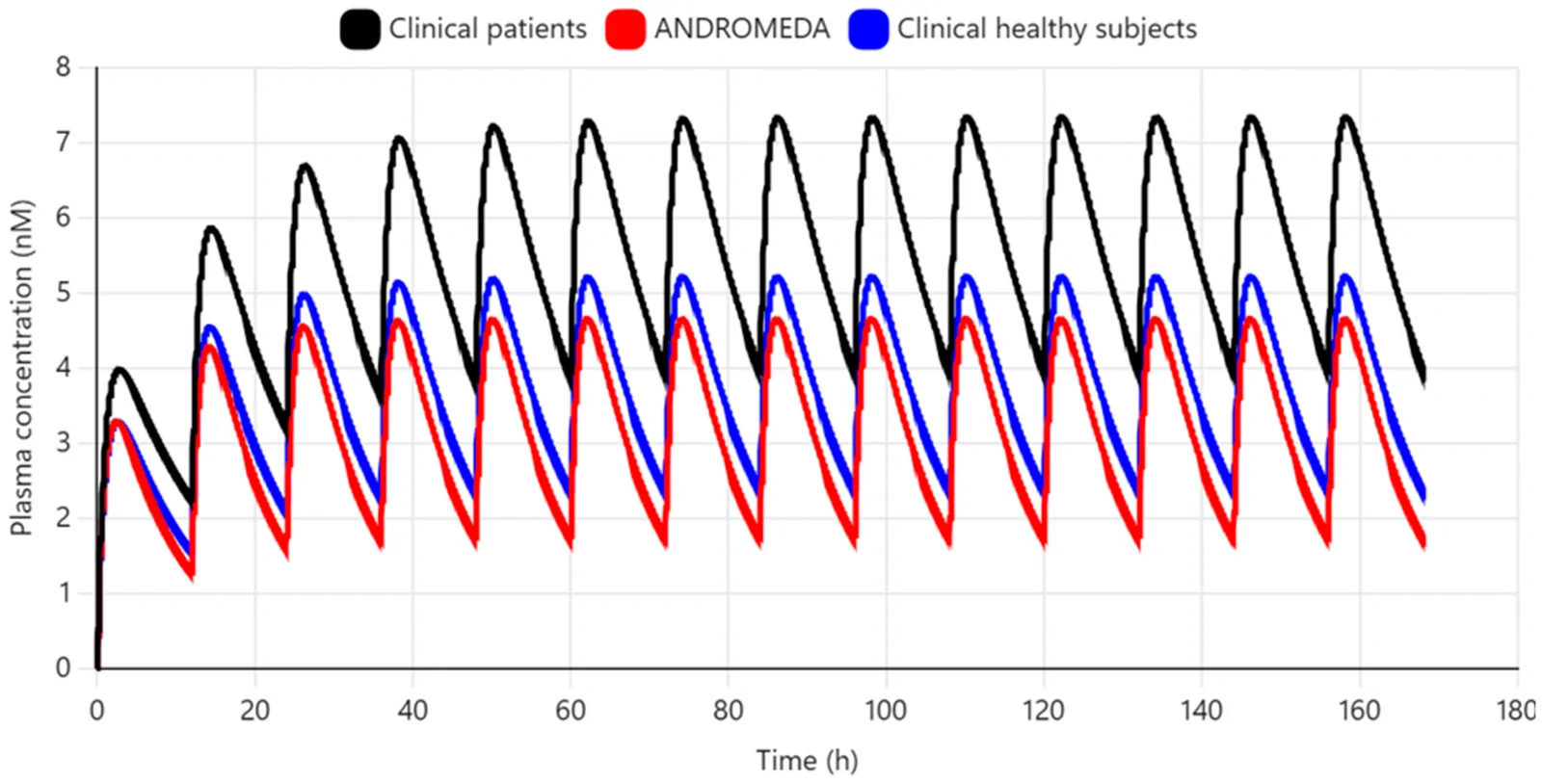
Predicted and clinically observed concentration–time profile of dofetilide (0.5 mg) administered orally twice daily for 7 days. The red line represents the ANDROMEDA predictions. The black line represents a model-based analysis of observed clinical patient data. The blue line represents observed clinical data from healthy subjects.

Following in silico target identification and affinity prediction, ANDROMEDA (version 2.0) was used to estimate pharmacokinetic (PK) profiles and drug exposure for verapamil and dofetilide under different dosing regimens [14,15,16,17,18]. Combining predicted drug exposures with drug-target affinity estimates enables an assessment of the expected level of target engagement in vivo. The structure-based predicted concentration–time profiles of oral dofetilide (0.5 mg) administered twice daily for 7 days is shown in Figure 5. The prediction was compared with clinical parameter estimates, obtained from previously published literature, of a population PK model in a patient with a body weight of 70 kg [19]. In this model, based on patient data reported in the drug label [20], oral bioavailability (F) was 0.9, apparent oral clearance (CL/F) was 16.2 L/h, the apparent volume of distribution (V/F) was 233 L, and the first-order absorption rate constant (k_a_) was 1 h^−1^. Andromeda predictions of CL/F, apparent volume of distribution at steady-state (V_ss_/F), and k_a_ of 28.9 L/h, 262 L, and 1 h^−1^, respectively, resulted in an underprediction of drug exposure compared with the clinical model-based patient data. However, using a clinical CL/F value of 24.2 L/h and a V/F of 272 L based on the CL of 23 L/h and V of 245 L reported in healthy subjects after intravenous administration [21], observed and predicted concentration–time curves were similar (Figure 5).

ANDROMEDA also provided predictions of plasma protein binding and fraction unbound (f_u_) drug. For dofetilide, f_u_ was predicted at 0.31. This is to be compared with the data in the label, describing a plasma protein binding of 0.6–0.7 [20]. A full comparison between the verapamil and dofetilide predicted PK parameters and reported estimates [22,23] are summarized in Table 1.

### 2.4. Emax Curve-Fitting Tool

**Figure 6:**
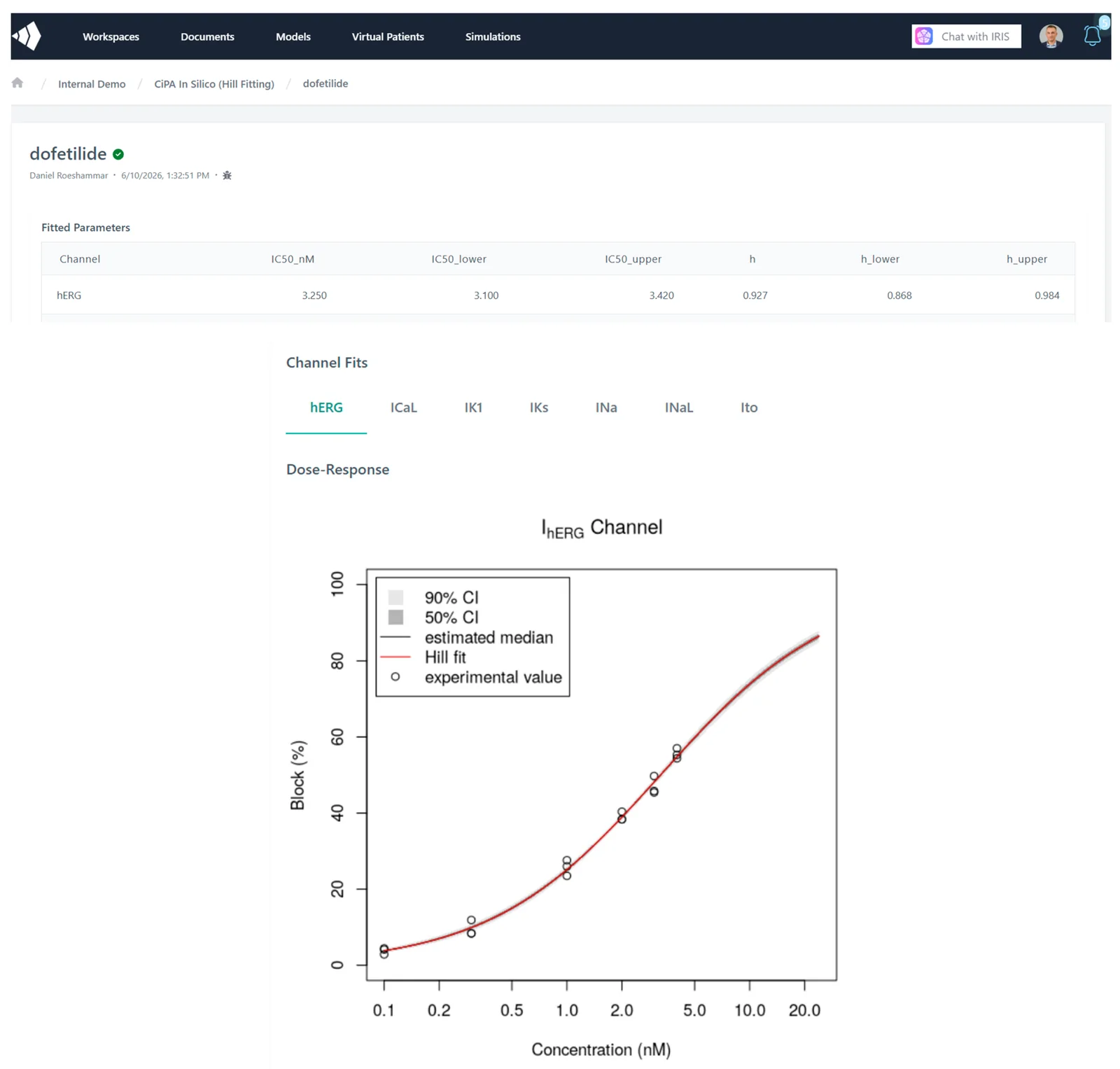
Emax curve-fitting tool results dashboard for dofetilide hERG channel inhibition based on in vitro data.

For selected compounds, to confirm the presence or absence of risk, ion channel inhibition should be estimated using in vitro experimental data. The E_max_ curve-fitting function (https://github.com/FDA/CiPA/tree/master, accessed on 12 October 2026) was applied to in vitro hERG inhibition data for dofetilide reported by Li et al. [24]. Users can upload raw in vitro experimental data in comma-separated value format, after which drug effect parameters are automatically estimated. In this example, the half-maximal inhibitory concentration (IC_50_) for hERG inhibition was estimated at 3.25 nM with a 95% CI of 3.10–3.42 nM (Figure 6). The resulting parameter estimates can subsequently be used as inputs for mechanistic in silico models and quantitative proarrhythmic risk assessments and QT liability.

### 2.5. Benchmarking Based on Competitor Data

**Figure 7:**
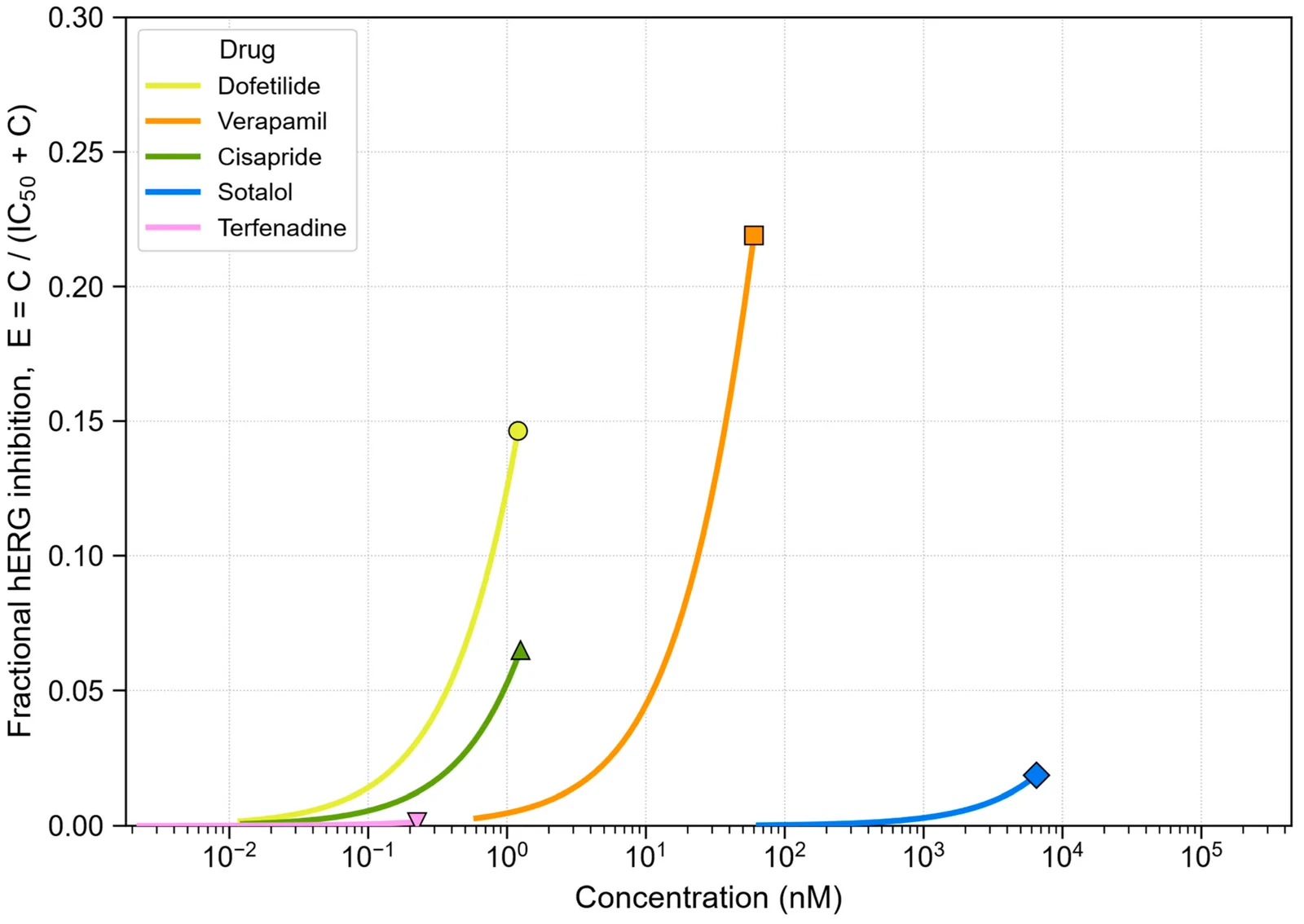
Benchmarking of fractional hERG channel inhibition by drug concentration for dofetilide, verapamil, cisapride, sotalol, and terfenadine. The unbound maximum drug concentration is marked with symbols for each drug.

To gain an understanding of how a new compound’s affinity is positioned relative to other drugs acting on the same target, either publicly available data or in-house reference libraries can be leveraged. Using our previously proposed benchmarking framework [25], developed on the basis of the DICTrank dataset [26], we quantitatively compared hERG target engagement across a set of drugs. Dofetilide, verapamil, cisapride, sotalol, and terfenadine have been reported to inhibit hERG channels with in vitro IC_50_ estimates of approximately 7 nM, 214 nM, 18 nM, 343 uM, and 165 nM, respectively [27]. These values are consistent with the results previously reported by Redfern et al. [28]. To relate hERG potency to clinical exposure, IC_50_ estimates were compared with unbound maximum plasma concentrations (C_max_) of approximately 1.2 nM, 60 nM, 1.25 nM, 6500 nM, and 0.225 nM, respectively [28]. When visualized within our benchmarking framework, both verapamil and dofetilide exhibit substantial target engagement relative to, e.g., cisapride, a drug associated with clinically significant QT interval prolongation that was ultimately removed from the market due to an unfavorable benefit/risk profile (Figure 7).

### 2.6. QT/TdP Risk Screen Tool

**Figure 8:**
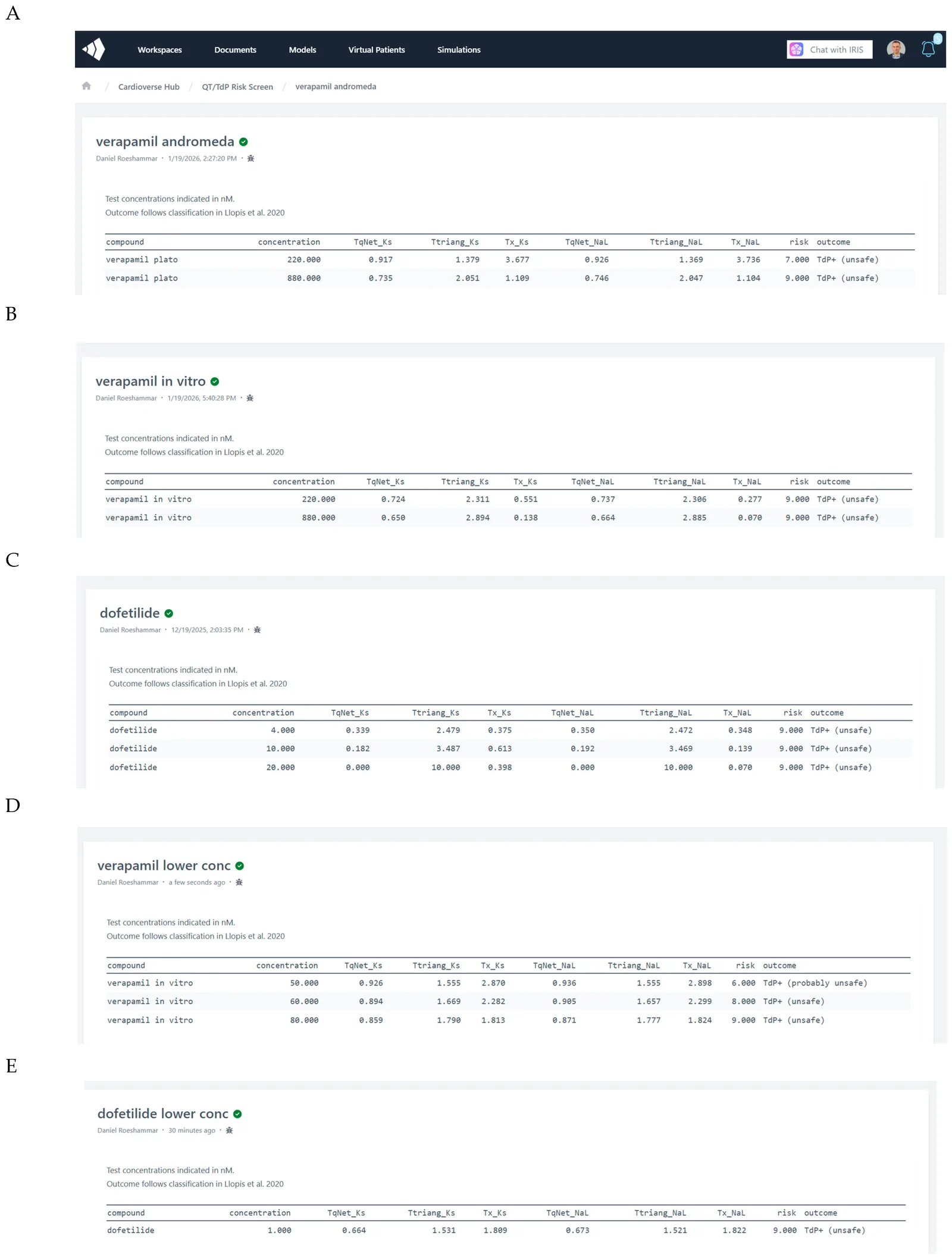
Results dashboard from the QT/TdP tool for verapamil based on PLATO prediction of target affinity (**A**) and in vitro data (**B**) as well as for dofetilide based on reported in vitro data (**C**). To account for plasma protein binding, lower drug concentrations were also tested for verapamil (**D**) and dofetilide (**E**). Outcome follows classification in [30].

To translate molecular structure-based and/or in vitro-derived target affinity into prediction of QT prolongation risk, we employed in silico models. The QT/TdP tool (version 1) [29,30] predicted a high risk of QT prolongation for verapamil based on PLATO-predicted target affinities (Figure 8A) and experimentally determined in vitro IC_50_ estimates of 499 nM for hERG and 202 nM for calcium channels [31] (Figure 8B). Similarly, a high QT risk was predicted for dofetilide based on an in vitro IC_50_ value of 4.9 nM [31] (Figure 8C). When applying lower test concentrations, accounting for plasma protein binding, the risk score was reduced for verapamil (Figure 8D) but not for dofetilide (Figure 8E). While the dofetilide prediction is consistent with its known effects in humans, the prediction for verapamil appears to overestimate its QT prolongation risk. The discrepancy may reflect this reduced model’s limited ability to account for the opposing effects of concomitant calcium and other ion channel inhibition.

### 2.7. STrhiPS Tool

**Figure 9:**
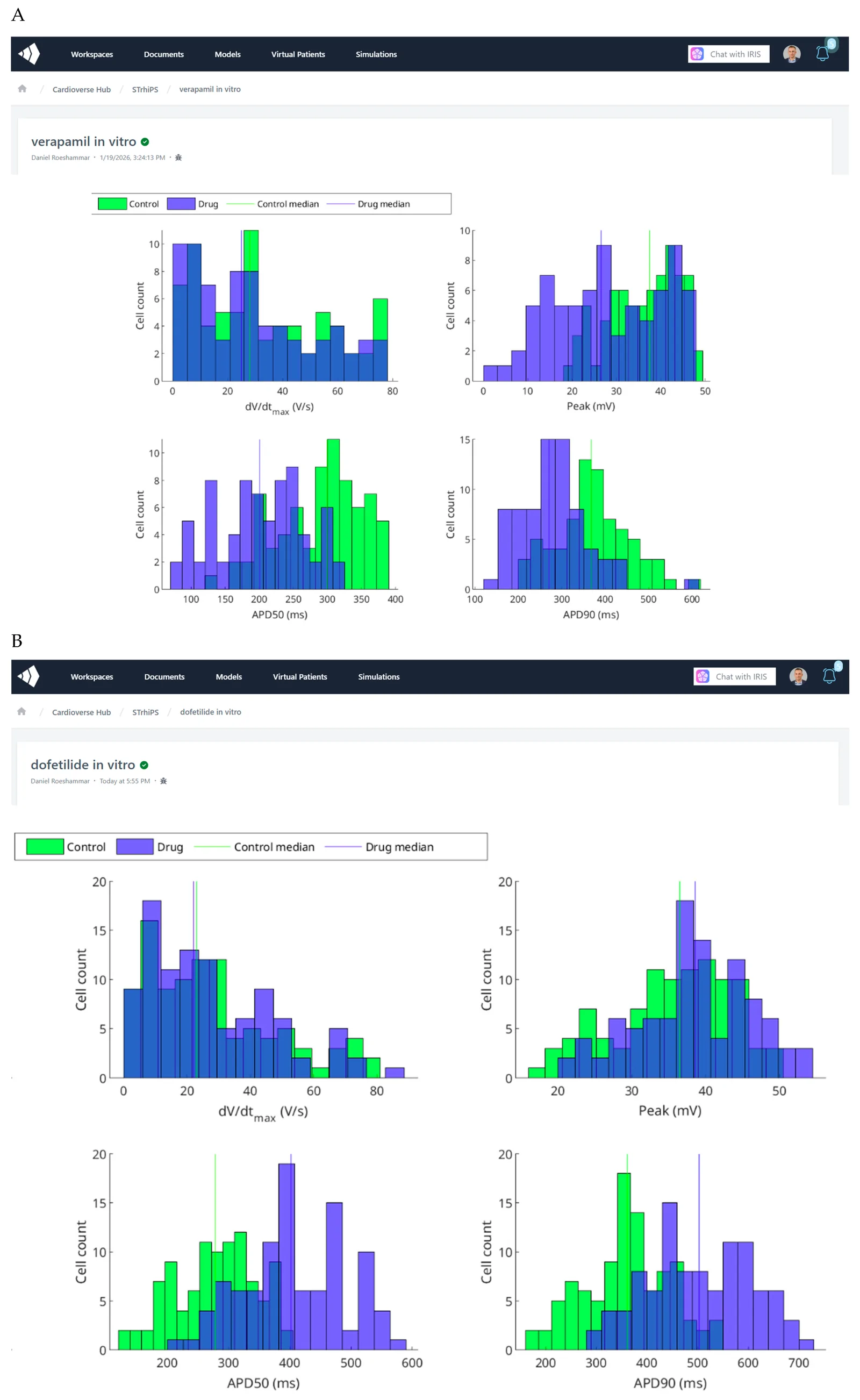
STrhiPS predictions of ion channel effects on dV/dt_max_, peak, APD_50_, and APD_90_ for verapamil (**A**) and dofetilide (**B**) based on in vitro data (100 simulated cell replicates). The blue and green bars show the results with and in absence of drug, respectively.

The STrhiPS (Safety Trials tool based on human-induced Pluripotent Stem cells) tool (version 4) provided a more comprehensive assessment of QT prolongation risk [32,33]. For verapamil, the model predicted no increase or even a reduction of the action potential duration at 90% repolarization (APD_90_), a surrogate marker for QT, using in vitro IC_50_ values of 499 nM and 202 nM for hERG and calcium channels, respectively [31] (Figure 9A). In contrast, for dofetilide, the model predicted a concentration-dependent increase in APD_90_ relative to the control using an in vitro IC_50_ estimate of 4.9 nM [31] (Figure 9B). For verapamil, the predicted change in APD_90_ relative to the control was −90 ± 56 ms and −139 ± 90 ms at concentrations of 220 nM and 880 nM, respectively. For dofetilide, the corresponding change was 71 ± 23 ms and 146 ± 51 ms at concentrations of 2 and 5 nM, respectively.

### 2.8. CiPA-PK Tool

The CiPA-PK tool provides a comprehensive framework for predicting QT risk by integrating in vitro ion channel data with PK predictions [19]. This approach illustrates how in vitro data and PK predictions can be translated into clinically relevant dosing recommendations. For dofetilide, the authors demonstrated that the standard dose of 0.5 mg twice daily administered to women with renal impairment could result in substantial prolongation of APD_90_ and induce repolarization abnormalities in 10 of 300 simulated models. When the dose was reduced to 0.125 mg twice daily, in accordance with the approved prescribing label information, APD_90_ decreased from 545 ms to 431 ms, and the incidence of repolarization abnormalities was reduced to zero.

### 2.9. Cardiac Emulator

The Cardiac emulator represents a novel and promising approach for translating in vitro findings into predictions of clinical QT effects, providing a complementary approach to translational predictive frameworks such as the Cardioverse suite [31,34]. The Cardiac emulator has been reported to predict concentration–QT relationships that were in close agreement with clinical observations for both verapamil and dofetilide. For verapamil, the model predicted a critical total concentration of 246 ng/mL (232 ng/mL in females, 259 ng/mL in males) (equivalent to 541 nM) based on in vitro derived input, compared with an estimate of 232 ng/mL based on clinical data. However, it should be noted that the verapamil results were not corrected for heart rate. For dofetilide, the model predicted a critical total concentration of 0.38 ng/mL (0.36 in females and 0.39 in males) (equivalent to 0.86 nM) based on in vitro computations, compared with an estimate of 0.41 ng/mL based on clinical data [34].

### 2.10. PKPD Model for Animal QT Studies

Semi-mechanistic pharmacokinetic-pharmacodynamic (PKPD) models are available to characterize drug-specific effect parameters from cardiovascular response measurements obtained in animal studies. Here, we used an indirect-response turnover model with feedback systems driven by changes in heart rate. Reported applications include analyses of cardiac safety parameters, including QT prolongation of dofetilide in rats [35] as well as analyses for verapamil and dofetilide in dogs [36]. In these analyses, verapamil was found to stimulate the zero-order production rate of heart rate (resulting only in indirect effects on uncorrected QT), whereas dofetilide was found to directly stimulate the turnover rate of QT (without affecting heart rate).

**Figure 10:**
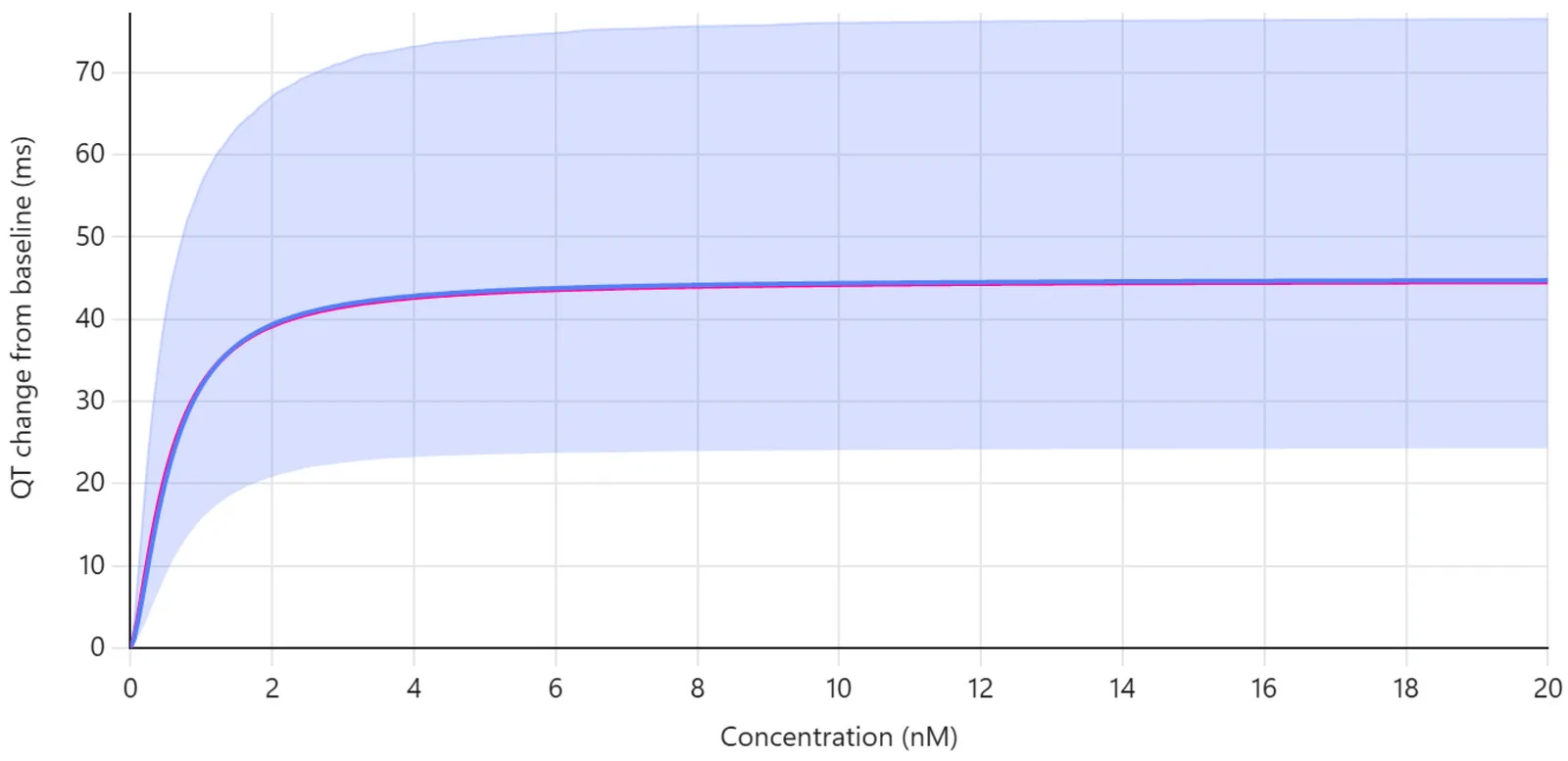
Translated dofetilide concentration–response relationship to humans based on scaling from dog data. The figure shows a simulation of 10 virtual subjects. The solid lines show the nominal model and the mean response. The shaded area is a 95% prediction interval across the 10 individual predictions.

The dofetilide E_max_ (describing the maximal proportional increase in the QT turnover rate), unbound EC_50_ and hill factor were reported at 0.097, 0.561 nM, and 1.54, respectively. Baseline QT in dogs was reported at 238 ms. In humans, we assumed a heart rate-corrected QT baseline of 450–460 ms. After accounting for a human plasma protein binding of 35–40% [28] and assuming a similar human EC_50_ value, simulations of the expected clinical concentration–QT response curve at steady-state are shown in Figure 10. Between-subject variability of 30 CV% was explored on both the EC_50_ and baseline QT estimates in simulations with 10 virtual subjects. At the expected therapeutic total C_max_ of approximately 2 nM [28], the average QT increase was predicted at 40 ms relative to baseline.

The model can be applied to longitudinal single or multiple dose, repeatedly measured QT data to quantify specific drug effect parameters. The model can be used for both design and interpretation of animal studies. Importantly, the model can be used to explore the impact of clinical study design on the expected outcomes in humans.

### 2.11. PKPD Translational Tool

When simulation of the expected clinical QT time course and prediction of peak clinical drug concentrations under different dosing regimens are required, the PKPD translational tool can be used [37]. The tool enables translation of PKPD information from preclinical species to humans by accounting for differences in body weight and plasma protein binding. For example, PK parameters estimated in dogs can be extrapolated to humans and compared with independent predictions generated using ANDROMEDA.

**Figure 11:**
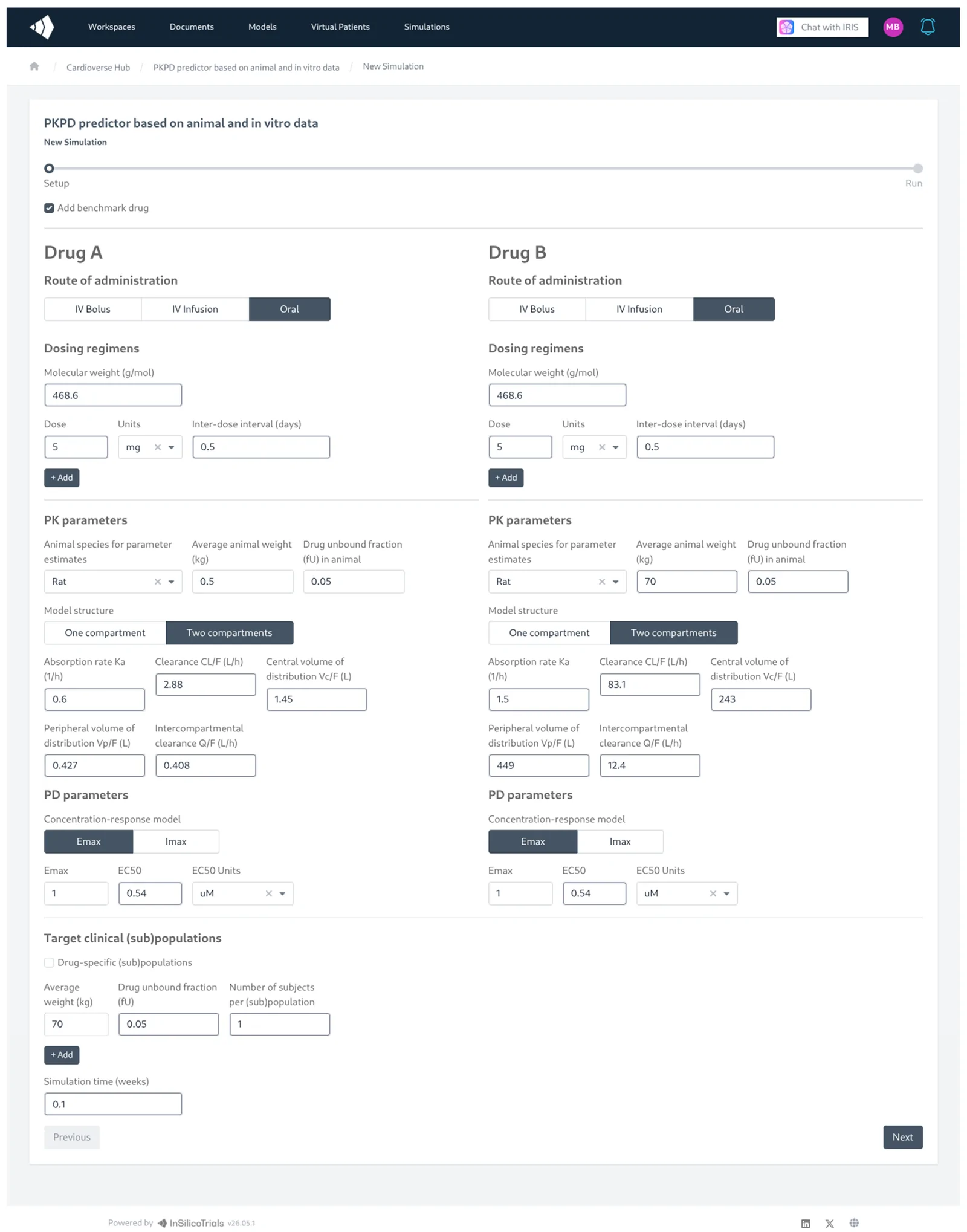
Input wizard of the PKPD translational tool, enabling clinical predictions based on PK and drug effect parameters from molecular structure predictions, in vitro assays, or animal or clinical data.

Users specify the intended dosing regimen(s) together with relevant PK and drug effect parameters obtained from molecular structure, in vitro assays, or animal studies. Additional inputs include species-specific plasma protein bindings and body weights. The tool can also be used to scale drug exposure and response from adult to pediatric populations, supporting pediatric study planning and dose selection. A snapshot of the input wizard is presented in Figure 11. For advanced technical users, the platform also supports the integration of more mechanistic models through direct incorporation of user-specified code, including NONMEM models [38] and models implemented using the mrgsolve package [39].

### 2.12. Clinical Concentration–QT Estimation Tool

**Figure 12:**
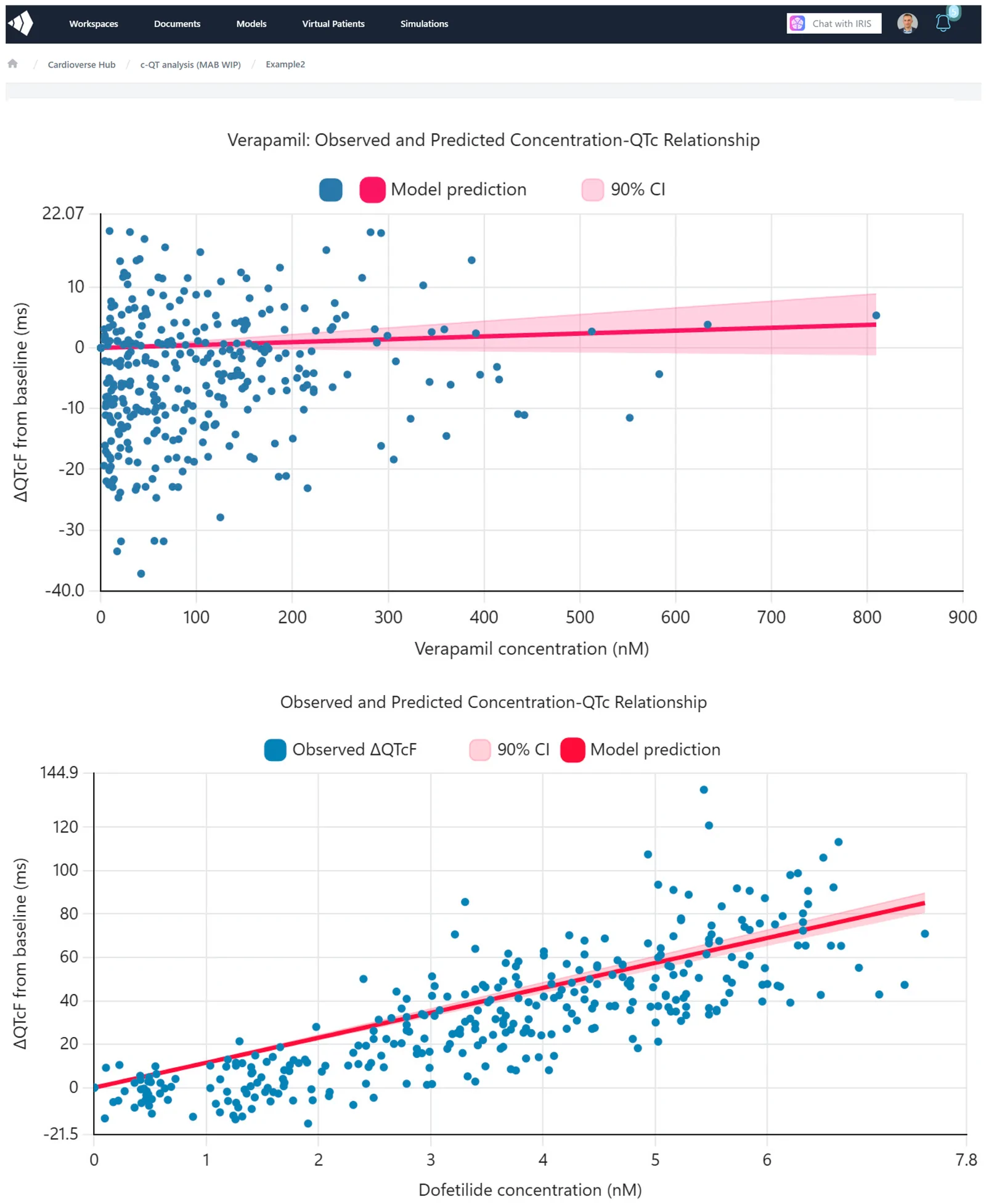
Observed and model predicted concentration–QT relationship in humans. Blue symbols represent observed data. The red shaded area is a 90% CI around the mean predicted effect.

Once clinical data become available, the drug effect should be characterized using concentration–response modeling to ensure that any QT effects remain within acceptable safety limits. Using the clinical concentration–QT estimation tool [40] with the original data from Johannesen et al. [41], we estimated the concentration–response relationship for verapamil and dofetilide on the platform. Users can upload raw ECG results from early-phase clinical studies, including Phase I SAD and MAD or TQT studies. The tool then automatically performs data visualization and concentration–QT modeling, generating key drug effect parameter estimates and diagnostic output. The framework can be used to quantify the probability that the maximum therapeutic drug exposure in any population or subgroup of risk will exceed predefined limits, such as the 10 ms QT prolongation safety threshold. Based on the observed clinical data, verapamil did not show a predicted QT prolongation of 10 ms or more at the upper bound of the 90% CI of the model predictions, using heart rate (Fredericia)-corrected data. In contrast, for dofetilide, the predicted 90% CI crosses the 10 ms safety threshold already at therapeutic drug concentrations (Figure 12).

## 3. Discussion

In this proof-of-concept framework, we demonstrated how QT interval prolongation risks can be predicted and quantified for novel drug candidates throughout the drug discovery and development process using an in silico computational framework. Through early prediction based on molecular structure, the potential risk for QT prolongation can be identified before experimental testing. This approach has the potential to reduce the number of costly in vitro studies on drugs with a high likelihood of failure. Importantly, the integration of in vitro data with in silico prediction methods enables more informed candidate selection, potentially reducing the risk of unsuccessful animal studies and supporting better-informed clinical development programs. Furthermore, we demonstrated how drug-induced effects on cardiac repolarization can be quantitatively assessed using both animal data and clinical data. Historically, such analyses required extensive specialist expertise and substantial manual efforts. At present, many of these analyses can be automated, enabling broader adoption and more consistent decision-making.

Integrating tools for QT risk assessment based on molecular structure, in vitro pharmacology, animal studies, and clinical data within a unified workflow provides a comprehensive framework for supporting dose justification and transparent regulatory assessments based on the totality of evidence. This learn and confirm [42] framework supports decision making at every stage of drug development, facilitating evidence-based go/no-go decisions and proper resource allocation. Consequently, compounds with a high probability of unacceptable QT liability can be identified early and discontinued before substantial development resources are committed.

The aim of the current conceptual work was not to validate each individual tool or establish definitive decision criteria for the complete workflow but rather to demonstrate the concept of integrating computational and predictive in silico approaches throughout the drug discovery and development process. By adopting a more holistic perspective and combining complementary methods across different stages of development, a stronger evidence basis can be generated for assessing the risk of QT prolongation. The proposed workflow illustrates how the integration of multiple lines of evidence can facilitate the early identification of compounds at risk and enhance confidence in predictions of risk associated with therapeutic drug exposures. Each individual tool provides uncertainty estimates, which can be incorporated in the subsequent predictions. For simplicity, only the point estimates were carried forward in the present application. When used for probabilistic decision-making, the precision in the results should be considered. The various tools may be used in sequence or in parallel depending on the availability of data. Sequential use of multiple predictive methods may propagate cumulative uncertainty throughout the work.

Using CUPID, a preliminary assessment of QT liability was performed based solely on molecular structure and predicted interactions with hERG, calcium, and sodium ion channels. The results indicated that verapamil has a high likelihood of interacting with both hERG and calcium channels due to structural alerts of the molecule. Similarly, dofetilide was predicted to have a high likelihood of interacting with hERG channels.

PLATO was subsequently used to quantify the expected affinity of these compounds for the relevant ion channels based solely on molecular structure. For verapamil, the predicted affinities for hERG and calcium channel inhibition, expressed as dissociation constants (K_D_), were 86.8 nM and 122.6 nM, respectively. Corresponding in vitro measurements have been reported as IC_50_ values of 214 nM [26] and 499 nM for hERG [31] and 202 nM for calcium [31]. Thus, PLATO predictions were generally within the same order of magnitude as the experimentally observed values, although reported on different but related scales. For dofetilide, PLATO predicted a hERG K_D_ of 84.2 nM. In vitro studies have reported substantially higher potency, with IC_50_ values of 7 nM [27] and 4.9 nM [31]. While this represents a greater than tenfold deviation from the reported experimental values, the predicted affinity still indicates a strong predicted interaction with hERG and therefore provides an early indication of potential QT liability. It should be noted that PLATO was developed to predict bioactivity across a broad range of pharmacological targets and is not restricted to cardiac ion channels. Consequently, predictive performance for individual targets may not be fully optimized for specific targets and cardiac safety applications. However, the underlying model can be retrained and customized to focus on a more restrictive set of ion channels and cardiac safety-related targets, potentially further increasing predictive accuracy.

ANDROMEDA was used to predict human PK profiles directly from molecular structure. Predicted drug concentration–time profiles showed good agreement with observed clinical data in healthy subjects, within the range of variability reported (27 CV% for CL and 22 CV% for V) [21]. These results demonstrate the potential of molecular structure-based prediction to provide early estimates of therapeutic exposure and safety margins at intended dose levels, potentially enabling assessment before animal or clinical data become available. It should be noted that model-based analyses of clinical data in patients showed higher exposure than the ANDROMEDA predictions and data in healthy subjects. This is analogous to the elevated risk observed in certain patient subgroups who experience increased drug exposure, for example, as a result of impaired liver or kidney function. For QT risk assessment, maximum drug exposure is of particular importance. However, for many pharmacological and safety endpoints, the entire concentration–time profile may be relevant to explore. In this context, the integration of PLATO and ANDROMEDA provides an unprecedented opportunity to predict target engagement and pharmacological effects as a function of time and dosing interval [37].

Using in vitro data, we illustrated how IC_50_ values for ion channel interactions can be rapidly estimated without requiring specialized computational expertise. By simply uploading raw experimental data, concentration–response relationships can be analyzed with potency estimates generated in an automated manner. Furthermore, we demonstrated how IC_50_ estimates, in combination with plasma protein binding and predictions of clinically relevant concentrations, can be benchmarked against drugs with known QT-prolonging effects.

By entering the IC_50_ values into tools such as QT/TdP, STrhiPS, CiPA-PK, and the Cardiac emulator, we demonstrated how in vitro ion channel potency data can be translated into predictions of QT prolongation risk. QT/TdP can be used for a rapid high-level assessment of potential pro-arrhythmic risk, making it well suited for early screening and prioritization. In contrast, STrhiPS provides a more detailed and quantitative evaluation, allowing a more mechanistic elucidation of predicted effects on cardiac repolarization. CiPA-PK further extends these analyses by incorporating PK information, enabling the evaluation of time-varying drug exposure over clinically relevant dosing intervals and across subgroups of patients in which drug exposure may be higher or lower due to expected covariate effects.

In the present example, the apparent misclassification of verapamil by QT/TdP may be attributable to several factors. The underlying modelling approach may not fully capture the complex pharmacological effect of verapamil on cardiac electrophysiology and account for the extent to which inhibition of calcium and other ion channels counteracts the effects of hERG inhibition. When the accuracies of four different TdP classifiers were compared, it was shown that 106 of 109 compounds, including verapamil, were correctly classified when seven ionic currents were considered. Using simplified approximations rather than the full electrophysiological model, predictions based on QT/TdP still demonstrated good accuracy, sensitivity, and specificity although a few drugs, such as verapamil, were identified as false positives [30]. In contrast, STrhiPS provided a more accurate prediction, reflecting the balance between the opposing effects of hERG and calcium channel inhibition. Any signal detected by QT/TdP should therefore be further scrutinized using tools such as STrhiPS and CiPA-PK.

Using the Cardiac emulator, which directly predicts QT interval effects rather than surrogate electrophysiological effects such as APD_90_, there was a good match between reported predictions based on in vitro data and clinical outcomes [31]. This approach should be routinely considered in modern drug development programs to accelerate decision-making and to decrease uncertainty. It may require further validation but seems to have the potential to gradually reduce the reliance on animal testing.

Using animal data, no direct QT prolonging effect of verapamil could be detected. For dofetilide, a concentration–response relationship could be detected using an indirect-response PKPD model [36]. Although we anticipate that traditional animal studies will increasingly be complemented, or in some cases replaced, by advanced in vitro and in silico approaches, PKPD modeling remains a valuable tool for quantifying drug effects in large animal studies. The result can rapidly be translated into the clinical setting by accounting for interspecies differences in body size and plasma protein binding [37].

Automated concentration–QT modeling of the clinical data confirmed that verapamil was not predicted to prolong the QT interval by ≥10 ms at clinically relevant concentrations. For dofetilide, the concentration–QT analysis identified the concentration at which the upper bound of the 90% CI exceeds the 10 ms threshold. This provides a quantitative estimate of the exposure associated with a clinically relevant QT effect and illustrates how concentration–QT modeling can be used to characterize cardiac safety margins and support dose selection.

Many of the tools used in this workflow are regularly updated and refined based on emerging new data and are managed through version control. As the platform supports versioned models, all computations are traceable and reproducible.

Historically, clinically relevant QT signals were often identified late in development or even after regulatory approval, resulting in costly delays, additional studies, label restrictions, or, in some cases, market withdrawal. The integration of nonclinical QT risk assessment and clinical concentration–QT modeling has substantially improved the early identification and characterization of QT liabilities, reducing the likelihood of unexpected findings during late-stage development. Clinical concentration–QT modeling based on data from early phase studies, when combined with an adequate preclinical safety package, can frequently replace the need for a dedicated thorough QT study. A European Medicines Agency (EMA) review of Scientific Advice requests involving QT-related questions demonstrated this trend. Among 110 requests, 81% sought QT waivers, most justified by concentration–QT modeling. Of these requests, 70% were endorsed and a further 9% were conditionally endorsed. Common reasons for rejection included insufficient exposure margins, limitations in study design and gaps in the supporting data package [43].

Similar trends have been observed by the US Food and Drug Administration (FDA). Reviews of study report reviews between 2016 and 2024 demonstrated a 34% reduction in the number of TQT studies, accompanied by the increased use of concentration–QT modeling. In parallel, the incorporation of an integrated nonclinical risk assessment increased substantially from 2020 to 2024 [44].

With this conceptual framework, we hope to promote even earlier detection and characterization of QT liability, ultimately reducing the frequency of unexpected QT findings in clinical development as well as in animal studies. By making these tools readily accessible and easy to apply by multidisciplinary project teams, QT risk assessment can increasingly be shifted to earlier stages of drug discovery and development. As a consequence, Phase I studies may evolve from primarily exploratory investigations of QT risk toward confirmatory studies designed to verify predictions generated from integrated nonclinical and translation assessments. Even for drugs where QT prolongation is considered acceptable within a favorable benefit–risk profile, such as many oncology indications, these approaches can provide value for the early exploration of various dosing strategies and characterization of risk.

Similar tools and alternative approaches may be available, allowing the creation of multiple workflows tailored to different development needs. For example, in a recently proposed quantitative weight of evidence framework, additional methods for eliminating the detection of false positive drug candidates have been suggested with the aim of restoring the lost opportunity cost for abandoned medicines [45]. The objective of this work was not to advocate a specific workflow but rather to demonstrate the feasibility and value of applying in silico predictive methods from early discovery through clinical development. By leveraging such tools, the totality of evidence can be more clearly defined, quantitatively integrated, and interpreted in a consistent manner. The combined use of complementary methods has the potential to further streamline and strengthen the assessment of cardiac safety risks. Equally important, these methods can be collected within a unified framework, enabling the seamless integration of evidence across disciplines and development phases. While the examples presented here demonstrate the potential utility of this framework, further prospective evaluation using novel drug candidates is warranted. Such studies would enable a more comprehensive assessment of predictive performance, identify opportunities for refinement, and provide additional guidance on the optimal application of these methods in drug development and regulatory decision-making.

## 4. Materials and Methods

### 4.1. QT-Prolongation Risk Assessment Workflow

**Figure 13:**
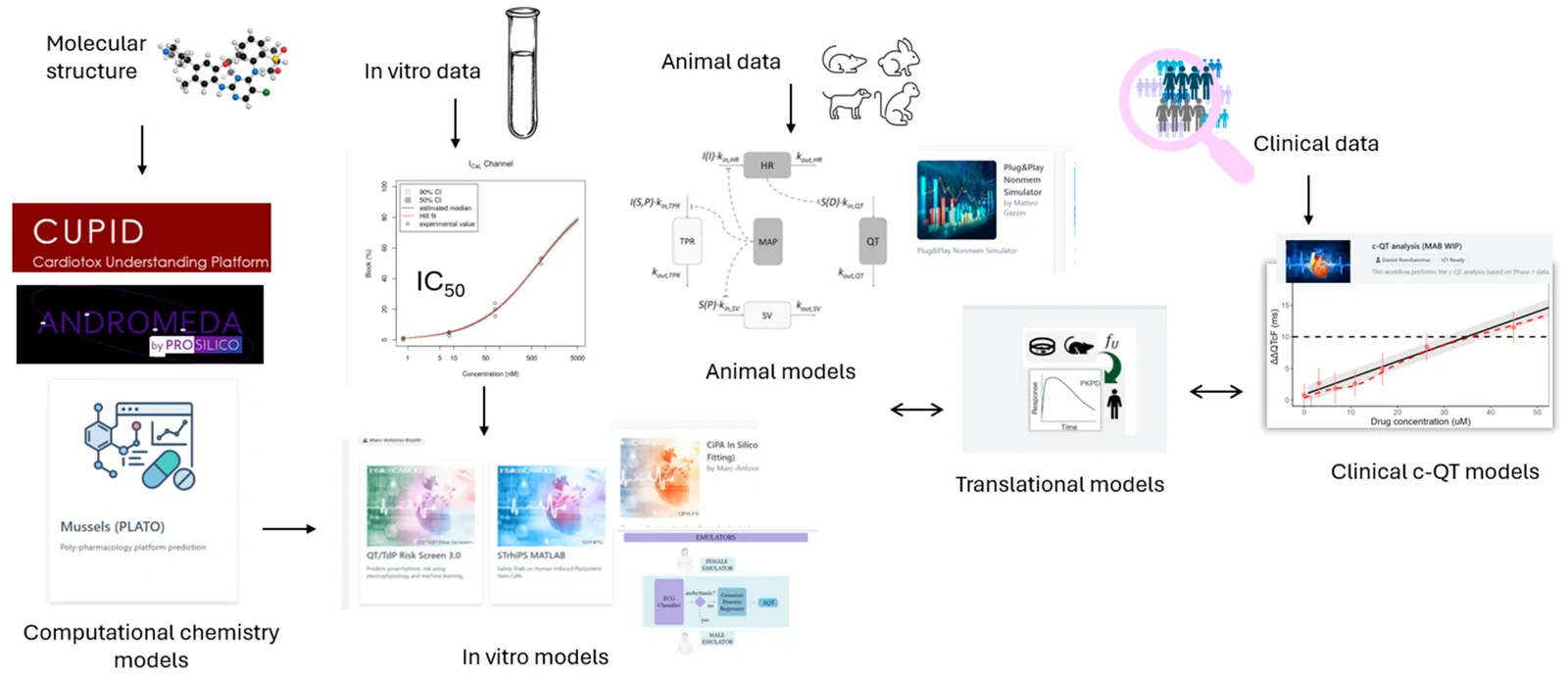
Overview of an in silico QT risk assessment framework with applications from discovery to clinical development. Based on molecular structure, in vitro, animal, and clinical data, the next experiment can be optimized and go/no-go decision making can be informed. Results from each experiment contributes to the totality of evidence for safety assessment and dose justification.

An overview of a conceptual representative quantitative QT risk assessment workflow is presented in Figure 13. Assessment of QT liability can begin at an early stage of drug discovery based solely on molecular structure. Users provide the simplified molecular-input line-entry system (SMILES) representation of a candidate compound, which is used to generate predictions of potential interaction with key cardiac ion channels, together with quantitative estimates of target affinity and PK properties. This exploratory assessment enables the identification of compounds with potential cardiac safety liabilities, allowing candidates with an unfavorable predicted risk profile to be deprioritized or redesigned before further investment is made in in vitro testing.

Subsequently, data generated from in vitro assays can be used to derive drug-specific effect parameters, which serve as input to in vitro-based in silico models. These models can further support prioritization of drug candidates for downstream evaluation. Based on such results, more targeted animal experiments, for example, in telemetered dogs, can be conducted. The resulting data can then be analyzed using semi-mechanistic PKPD models that quantitatively describe the relationship between drug exposure and QT interval changes, while simultaneously accounting for other cardiovascular safety endpoints, including heart rate and blood pressure.

Translational modeling approaches enable the extrapolation of findings from in vitro and animal studies to the clinical setting by accounting for interspecies differences in factors such as body size, plasma protein binding, and suitable dosing regimens. These methodologies support the prediction and optimization of clinical study design. Finally, emerging clinical data from Phase I studies can be evaluated using automated model-based workflows to quantify drug-induced QT effects and refine dose justification for later stages of development. The individual applications that comprise this proof-of-concept workflow are described in more detail in the following sections and in the Results.

### 4.2. Workflow Tools

An overview of in silico tools relevant to QT risk assessment is presented in Table 2.

### 4.3. Verapamil and Dofetilide Case Example Drugs

Verapamil is a non-dihydropyridine calcium channel blocker that exerts its pharmacological effects by inhibiting L-type calcium channels, thereby reducing calcium influx into cardiac and vascular smooth muscle cells. This action results in vasodilation, reduced myocardial contractility and decreased heart rate. Verapamil is indicated for the treatment of hypertension, angina pectoris, tachycardia, and atrial fibrillation [46,47]. Importantly, despite being an inhibitor of the hERG channel, verapamil is not associated with a clinically relevant QT interval prolongation and may even shorten the QT interval. Any observed apparent increase in the uncorrected QT interval is often secondary to verapamil-induced heart rate reduction. Consequently, heart rate-corrected QT (QT_c_) intervals should be used when assessing the electrophysiological effects of verapamil [48].

Dofetilide, in contrast, is a class III antiarrhythmic agent that selectively blocks the rapid component of the delayed rectifier potassium current (IKr) mediated by the hERG channel. It is indicated for the conversion to and maintenance of normal sinus rhythm in patients with atrial fibrillation or flutter. Dofetilide hence prolongs the QT interval [49].

These two drugs were selected as illustrative retrospective case examples to demonstrate the application of the proposed conceptual workflow across different stages of drug discovery and development. Specifically, we highlight how QT risk assessment can be informed using data derived from molecular structure, in vitro assays, animal studies, and clinical trials. The workflow should be independently validated and tested across a larger set of drugs, including prospective validation with new compounds.

## 5. Conclusions

The risk of QT interval prolongation can be predicted and quantified based on drug concentration using molecular structure, in vitro, animal, and clinical data. Through the appropriate application of in silico prediction methods and tools, investment decisions at each stage of drug development can be better informed, thereby minimizing the risk of failed studies. Based on the totality of evidence, informed go/no-go decisions for further development can be made, and potential label claims can be supported with quantitative assessments.

We conceptually demonstrate how various in silico tools for QT safety assessment can be integrated into unified workflows to facilitate efficient use and effective cross-functional communication through the different stages of drug development. These solutions may enable the prediction and estimation of potential QT interval prolongation effects from bench to bedside. The framework should be further validated across a larger number of drugs.

## Figures and Tables

**Table 1:** Andromeda predicted and clinically observed PK parameters for verapamil and dofetilide.

	Dofetilide			Verapamil		
	Observed	Predicted	Error (-Fold)	Observed	Predicted	Error (-Fold)
V_ss_ [L/kg]	3.30	2.09 */2.62	1.3/1.6 *	3.70	1.83 */2.94	1.3/2.0 *
f_u_	0.35	0.31/0.57 *	1.1/1.6 *	0.10 (0.06–0.28)	0.03/0.20 *	2.0 */3.3
CL [mL/min]	364	318/957 *	1.1/2.6 *	1260	528/1178 *	1.0 */2.4
CL/F [mL/min]	404	482/1063 *	1.2/2.6 *	6000	1760/8000 *	1.3 */3.4
k_a_ ** [1/h]		ca 0.5–1			ca 0.5–1	
Oral F [%]	0.9	0.7/0.9 *	1.0 */1,1	0.21 (0.10–0.38)	0.18 */0.30	1.2 */1.4
t_½_ [h]	8 (5–14)	3.2 */8.7	1.1/2.5 *	5 (2–8)	1.5 */5.9	1.2/3.0 *

* Obtained in cross-validation with older models with performance inferior to current ANDROMEDA models. ** Considering influence of intestinal efflux and metabolism on ka. V_ss_: volume of distribution at steady-state, f_u_: fraction unbound, CL: clearance, CL/F: apparent oral clearance, k_a_: first-order absorption rate constant, F: bioavailability, t_1/2_: half-life. Observed values were retrieved in the work by Varma et al. [22] and Lombardo et al. [23].

**Table 2:** QT risk assessment tools based on molecular structure, in vitro, animal, and clinical data.

Tool	Category	Input Data	Core Functionality	Application	References
CUPID	Structure-based QT risk	Molecular structure (SMILES)	Explainable AI model predicting cardiotoxicity via ion channel interactions (hERG, Na, Ca); identifies structural alerts	Early prediction of QT risk and ion channel liabilities	[9]
PLATO	Structure-based target affinity	Molecular structure (SMILES)	Predicts drug–target affinities across ion channels, receptors, enzymes, and transporters	Target profiling, identification of off-target risks including ion channels	[10,11,12,13]
ANDROMEDA	PK/ADME prediction	Molecular structure (SMILES). Can be combined with in vitro and in vivo data	Predicts ADME properties and human pharmacokinetics	Estimates clinical exposure following various dosing regimens. Longitudinal prediction of target engagement over time when combined with PLATO	[14,15,16,17,18]
Emax fitting tool	In vitro data analysis	In vitro concentration–response data	Automated curve fitting with uncertainty estimation	Drug effect parameters (E_max_, IC_50_) for modeling	* [24]
Benchmark framework	Benchmarking	Molecular structure or in vitro data	Compares candidate drug effects to known drugs using FDA cardiotoxicity datasets	Quantitative risk benchmarking against existing compounds	[25,26]
QT/TdP	In vitro → risk prediction	Ion channel IC_50_ values	Rapid evaluation of QT/TdP liability based on potency data	General QT risk classification	[29,30]
STrhiPS	Mechanistic electrophysiology	Ion channel potency data	Predicts action potential duration (APD_90_) as a surrogate for QT interval	Mechanistic QT risk estimation	[32,33]
CiPA-PK	In vitro + PK integration	Ion channel data + PK predictions	Integrates electrophysiology and PK to simulate QT risk dynamically	Time-dependent QT/TdP risk over dosing interval	[19]
Cardiac Emulator	In silico electrophysiology	Ion channel data	Simulates QT interval directly using mechanistic models	Direct prediction of QT effects	[31,34]
Animal PKPD model	Nonclinical modelling	Animal PK and QT/HR data (rats and dogs)	Semi-mechanistic indirect-response models with heart rate feedback	Quantification of drug effects (and study design support	[35,36]
PKPD translational tool	Translational modelling	Molecular, in vitro, or animal data	Scales drug effects to humans accounting for body size and plasma protein binding	Clinical prediction of efficacy/safety endpoints	[37]
Concentration–QT tool	Clinical data analysis	Clinical ECG + PK data (Phase I)	Exposure–response modeling of QT interval	Quantification of QT effects and regulatory assessment	[40]

ADME: absorption, distribution, metabolism and excretion, APD_90_: action potential duration at 90% repolarization, Ca: calcium, ECG: electrocardiogram, E_max_: maximal drug effect, HR: heart rate, IC_50_: concentration at which half the maximal effect occurs, Na: sodium, PK: pharmacokinetics, SMILES: simplified molecular-input line-entry system, TdP: torsades de pointes. * Accessed at https://github.com/FDA/CiPA/tree/master, accessed on 12 October 2026.

## Data Availability

The original contributions presented in this study are included in the article. Further inquiries can be directed to the corresponding author.

## Abbreviations

The following abbreviations are used in this manuscript:

ADME	Absorption, distribution, metabolism, excretion
APD_90_	Action duration potential at 90% repolarization
Ca	Calcium
CI	Confidence interval
CiPA	Comprehensive in vitro proarrhythmia assay
CL	Clearance
CL/F	Apparent clearance
C_max_	Maximal concentration
CV	Coefficient of variation
EC_50_	Concentration at which the half maximal response occurs
ECG	Electrocardiogram
EMA	European medicines agency
E_max_	Maximal drug effect
F	Bioavailability
FDA	Food and drug administration
F_u_	Fraction unbound
HR	Heart rate
IC_50_	Half-maximal inhibitory concentration
ICH	International council for harmonization
K_a_	First-order absorption rate constant
K_D_	Dissociation constant
MAD	Multiple ascending dose
Na	Sodium
QSAR	Quantitative structure-activity relationship
PK	Pharmacokinetics
PKPD	Pharmacokinetic-pharmacodynamic
SAD	Single ascending dose
SMILES	Simplified molecular-input line-entry system
TdP	Torsades de pointes
TQT	Thorough QT
V	Volume of distribution
V/F	Apparent volume of distribution
V_ss_	Volume of distribution at steady-state
